# P010. OnabotulinumtoxinA for treatment of chronic migraine: results after 1 year of treatment

**DOI:** 10.1186/1129-2377-16-S1-A110

**Published:** 2015-09-28

**Authors:** Eleonora Rossi, Chiara Lupi, Silvia Benemei, Pierangelo Geppetti, Francesco De Cesaris

**Affiliations:** SOD Centro Cefalee e Farmacologia Clinica AOU Careggi, Dipartimento di Scienze della Salute, Università degli Studi di Firenze, Florence, Italy

OnabotulinumtoxinA (OnabotA) was approved for treatment of chronic migraine (CM) following the PREEMPT trials[1, 2], and was licensed in Italy in February 2013. Thus, in December 2013 we began to offer OnabotA to patients with CM who did not respond to other preventive therapy (mostly topiramate, beta blockers, amitriptyline and calcium channel antagonists). Demographic and migraine characteristics of patients were recorded, and changes in monthly migraine days and headache intensity (the latter variable was evaluated only after the first session of treatment), as well as, the number of symptomatic medications intake, were assessed. From December 2013, 40 patients (9 males, 31 females) were treated with at least four therapeutic sessions. Their mean age (± SD) at treatment onset was 51.3±12.6 years (range: 27-80). All the patients fulfilled the ICHD-III criteria for CM[3] and 28 (70%) were receiving other preventive therapy. In 34 of these patients (85%) overuse of symptomatic medications was observed at the onset of treatment. After obtaining an informed consent, we administered OnabotA according to the PREEMPT protocol (155 UI for a total of 31 injections divided across 7 head/neck muscles), performing no additional injections in the first session (from the second session we also adopted “follow the pain” protocol), and we gave the patients a paper diary to register the headache evolution. After one year of treatment we analyzed the data related to the 40 patients, observing positive results in all the variables considered. In particular, following 1-year treatment there was a reduction from 25.1±6.1 to 19.8±9.1 in the mean (± SD) number of monthly migraine days (reduction of 21%, p < 0.0001). The intake of symptomatic medications was reduced of about 12%, but the difference was not statistically significant. Most (78%) of the patients (n = 23) who had accurately registered the data reported that the greatest clinical benefit of OnabotA was the reduction in the headache intensity. Finally, no severe adverse event was observed, and mild pain in the injection site was the most reported experience. No patient discontinued the treatment because of side effects. We can conclude that the use of OnabotA according to the PREEMPT paradigm is an effective and well-tolerated treatment in patients with chronic migraine in a real-life setting.

Written informed consent to publication was obtained from the patient(s).
